# Tropical deforestation is associated with considerable heat-related mortality

**DOI:** 10.1038/s41558-025-02411-0

**Published:** 2025-08-27

**Authors:** C. L. Reddington, C. Smith, E. W. Butt, J. C. A. Baker, B. F. A. Oliveira, E. I. Yamba, D. V. Spracklen

**Affiliations:** 1 Institute for Climate and Atmospheric Science, School of Earth and Environment, University of Leeds, Leeds, UK; 2 Fiocruz Regional Office of Piauí, National School of Public Health, Oswaldo Cruz Foundation, Teresina, Brazil; 3 Department of Meteorology and Climate Science, Kwame Nkrumah University of Science and Technology, Kumasi, Ghana

**Keywords:** Environmental health, Environmental impact

## Abstract

Tropical deforestation induces local warming and is a potential human health risk, having been linked to elevated human heat stress and reduced safe outdoor working hours. Here we show deforestation-induced local warming is associated with 28,000 (95% confidence interval: 23,610–33,560) heat-related deaths per year using a pan-tropical assessment. Analysis of satellite data shows tropical deforestation during 2001–2020 exposed 345 million people to local warming with population-weighted daytime land surface warming of 0.27 °C. Estimated heat-related mortality rates are greatest in Southeast Asia (8–11 deaths for every 100,000 people living in deforested areas) followed by tropical regions of Africa and the Americas. In regions of forest loss, local warming from deforestation could account for over one third of total climate heat-related mortality, highlighting the important contribution of tropical deforestation to ongoing warming and heat-related health risks within the context of climate change.

## Abstract (web summary)

The authors assess the impacts of tropical deforestation and its subsequent local warming on human heat-related mortality. They estimate that deforestation-related warming (+0.27 °C) is associated with approximately 28,000 heat-related deaths per year.

## Main

Over recent decades, tropical forests have faced extensive deforestation and degradation^1,2^, driven primarily by agricultural expansion and logging^3^. The impacts of tropical forest loss are profound, affecting biodiversity^4^, global climate^5,6^ the hydrological cycle^7^ and human communities^8–12^. Previous studies have demonstrated a strong association between tropical forest loss and increases in surface temperature on both local^6,12–19^ and regional^20,21^ scales. The local warming associated with deforestation can be immediate and of substantial magnitude^22^, equivalent to or larger than that projected from a century of global climate change under a high emissions scenario^12,20^.

Human exposure to elevated temperatures presents a major potential health risk. Heat stress can negatively affect mood and mental health^23^, impair physical performance^24^ and reduce labour productivity^25,26^. Furthermore, exposure to heat is associated with an increased risk of morbidity and mortality from cardiovascular diseases and other causes^27,28^. Considerable heat-related mortality has been attributed to recent climate change^29^.

The effects of climate change on human health are compounded by socioeconomic and demographic factors^30^. Population vulnerability to climate change is linked to health expenditure and proportions of obese and elderly populations^31^. Along with these factors, heat-related human health effects can be modulated by the degree of technological adaptation^32^, which can be strongly linked to socioeconomic status^33^. In lower-income countries with limited adaptive capacity, including many countries in the tropics, heat-stress-related labour capacity losses may have substantial economic consequences^34^ and increase poverty^35^.

Tropical deforestation is associated with increases in human heat exposure^12,22,36^. In field experiments, deforestation-induced heat exposure has been demonstrated to reduce cognitive performance^37^ and labour productivity^26^. Across the tropics, local warming from deforestation between 2003 and 2018 was associated with losses in safe thermal working conditions for 2.8 million outdoor workers^12^. Little is known about the potential for deforestation-induced warming to lead to additional deaths at the pan-tropical scale. Wolff et al.^10^ showed that in Berau Regency in Indonesia, the combination of global climate change and deforestation between 2002 and 2018 increased population-weighted mean temperatures by 0.86 °C, accounting for an estimated 7.3–8.5% of all-cause mortality (or 101–118 additional deaths per year). Wider assessments of the population-weighted warming due to deforestation or the potential impacts on health and mortality are lacking but are urgently needed to inform land use policy and climate adaptation strategies.

Here, we make the first pan-tropical assessment of the population-weighted warming due to tropical deforestation and the associated heat-related mortality burden. We focus our analysis on tropical deforestation that occurred from 2001 to 2020. We use spatially explicit satellite datasets of annual tree cover change^1^ to identify forest loss at a ~1 km^2^ spatial scale. We use satellite datasets of land surface temperature^38^ to quantify surface warming that has occurred over this period. Through comparing land surface temperature change over locations of forest loss to neighbouring locations without forest loss, we isolate the warming due to deforestation. We then use human population distribution data^39^ to map population-weighted exposure to this warming. Finally, we use data on non-accidental mortality^40^ combined with relationships between heat exposure and excess mortality from the literature^31^ to estimate the heat-attributable excess mortality due to nearby tropical deforestation. Our analysis provides important evidence of the negative potential human health impacts of tropical deforestation at local, regional and national scales.

## Forest cover loss and local temperature changes

**Fig. 1:**
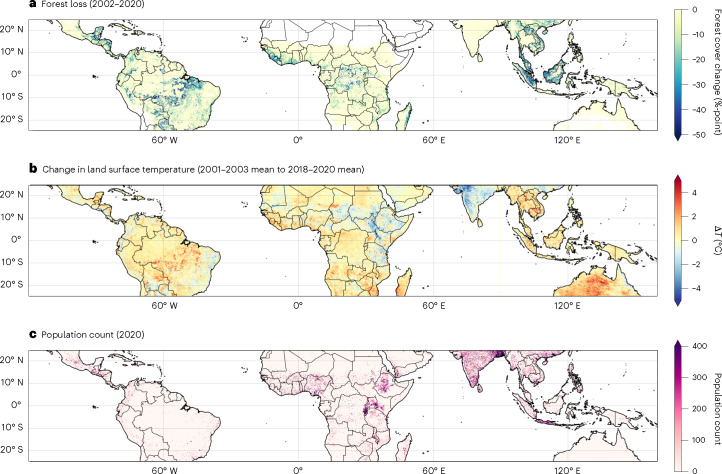
Forest loss, surface warming and population distribution across the tropics. **a**, The percentage-point change in forest cover from 2001 to 2020. **b**, The change in annual mean land surface temperature (Δ*T*) between the 2001–2003 mean and the 2018–2020 mean. **c**, The spatial distribution of population count in 2020.

During 2001–2020, a total of 1.6 million km^2^ of forest area (with greater than 10% forest canopy cover) was lost across the tropics. The greatest forest loss occurred across Tropical Central and South America (~760,000 km^2^), with extensive forest loss also occurring across Southeast Asia (~490,000 km^2^) and Tropical Africa (~340,000 km^2^). Surface temperatures in the tropics have generally increased over this time period due to a combination of global climate change and deforestation, with regional annual mean warming (Δ*T*) of +0.34 °C in Tropical Central and South America, +0.10 °C in Tropical Africa and +0.72 °C in Southeast Asia. Areas of forest loss coincide with areas of strong positive Δ*T* across many regions of the tropics (Fig. 1). We defined 1 km^2^ pixels that experienced at least two percentage points of forest loss during 2001–2020 as pixels that experienced deforestation (Methods). A total of 60% of pixels with warming of greater than four standard deviations from the mean (4.4 °C) experienced deforestation during 2001–2020, while deforested pixels make up only 17% of pixels in the tropics. The average Δ*T* in deforested locations across the tropics (+0.70 °C) exceeds warming that has occurred over areas that have maintained forest cover (+0.20 °C) (Table 1). Regionally, average Δ*T* in deforested locations (+0.73 °C in Tropical Central and South America, +0.75 °C in Tropical Africa and +0.61 °C in Southeast Asia) are 2–9× the average Δ*T* in locations that maintained forest cover (+0.30 °C in Tropical Central and South America, +0.08 °C in Tropical Africa and +0.31 °C in Southeast Asia). Similar to previous studies^10,12,17^, we assess the warming due to deforestation using land surface temperature data because near-surface air temperature data are not available at sufficiently high spatial resolution in the tropics. We compared land surface temperature and near-surface air temperature from the European Centre for Medium-Range Weather Forecasts Reanalysis v5 (ERA5 (ref. ^41^)) over the same period and find they agree to within 2% (Extended Data Table 1), confirming our approach is justified (Methods).

**Table 1:** Changes in annual mean land surface temperature (Δ*T*) in areas of the tropics that maintained (‘non-deforested’) and lost (‘deforested’) forest cover during 2001–2020

Region	Δ*T* in non-deforested locations (°C)		Δ*T* in deforested locations (°C)		Deforestation-induced local Δ*T* (°C)	
	Area-wgtd	Pop-wgtd	Area-wgtd	Pop-wgtd	Area-wgtd	Pop-wgtd
Tropical Central and South America	0.30	−0.12	0.73	0.40	0.53	0.22
Tropical Africa	0.08	0.12	0.75	0.97	0.39	0.32
Southeast Asia	0.31	0.36	0.61	0.56	0.37	0.26
Tropics (25° S to 25° N)	0.20	0.19	0.70	0.70	0.45	0.27

The estimated local Δ*T* due to forest loss between 2001 and 2020 (excluding the contribution of global climate change) is also shown (Methods). The area-weighted mean (‘area-wgtd’) and population-weighted mean (‘pop-wgtd’) Δ*T*s are shown for each region. All regions are bounded by latitudes of 25° S and 25° N.

## Deforestation-induced Δ*T* and human heat exposure

**Fig. 2:**
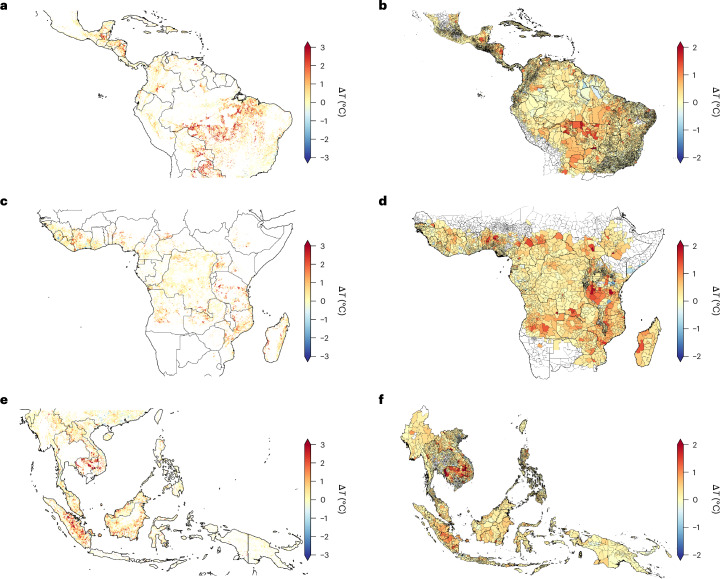
Change in local annual mean surface temperature (Δ*T*) due to deforestation during 2001–2020. **a**–**f**, The maps show regions of Tropical Central and South America (**a** and **b**), Tropical Africa (**c** and **d**), and Southeast Asia (**e** and **f**). The maps in **a**, **c** and **e** show the deforestation-induced Δ*T* at the ~1 km^2^ pixel level. The maps in **b**, **d** and **f** show the population-weighted mean deforestation-induced Δ*T* for second-level administration divisions for populations located in areas of forest loss. The division boundaries are from GADM (https://gadm.org/index.html). The areas with no data points are shown in white.

To estimate the Δ*T* due to deforestation only, excluding the contribution of global climate change and regional climate variability, we compared Δ*T* over locations of forest loss with neighbouring locations without forest loss. Over 2001–2020, deforestation is associated with 0.45 °C of warming on average across deforested areas of the tropics (Table 1). The regional mean deforestation-induced Δ*T* is greatest in Tropical Central and South America (+0.53 °C), with slightly lower Δ*T* in Tropical Africa (+0.39 °C) and Southeast Asia (+0.37 °C). This means that deforestation accounts for over half of the regional average warming in deforested areas of the tropics (and ~70% in Tropical Central and South America). In general, the regions with the greatest deforestation-induced warming correspond to the regions with the greatest forest loss (Fig. 2), particularly in the Arc of Deforestation in the southern Amazon and Sumatra and Kalimantan in Indonesia. Strong deforestation-induced warming is also visible in other areas that have experienced extensive forest loss^1^, including in Central America (Guatemala and Nicaragua), southern West Africa (Cote d’Ivoire, Ghana and Nigeria), Central and Eastern Africa (Democratic Republic of the Congo, Uganda and Tanzania) and Mainland Southeast Asia (Cambodia and Vietnam).

This local warming associated with deforestation has important implications for large populations that live across tropical forest regions (Fig. 1c). The tropics (25° S – 25° N) are home to over 3.5 billion people, with 13% (452 million people) living in regions that have experienced forest loss during the 2001–2020 period. We estimate that 345 million people were exposed to warming due to deforestation during 2001–2020 (Table 2), with substantial numbers of people exposed to deforestation-induced Δ*T* of greater than +1 °C (33 million people), +2 °C (8.0 million people) and +3 °C (2.6 million people). Across the tropics, 76% of people living in regions of tropical forest loss were exposed to deforestation-induced warming, with a similar proportion in the different tropical regions (74–80%). In Tropical Africa, 148 million people were exposed to warming from deforestation, compared with 122 million people in Southeast Asia and 67 million people in Tropical Central and South America.

**Table 2:** Population exposure to local warming from deforestation between 2001 and 2020 and the associated heat-related non-accidental mortality burden

Region/ country	Population in locations of forest loss	Population exposed to deforestation-induced warming	Annual deforestation-associated heat-related mortality	Annual deforestation-associated heat-related mortality rate (deaths per 100,000 people)	Percentage of total heat-related mortality	Percentage of total non-accidental mortality
Tropics (25° S to 25° N)	452 million	345 million (76%)	28,330 (23,610–33,560)	6 (5–7)	39.1%	1.06%
Tropical Central and South America	89.8 million	66.9 million (74%)	2,520 (2,160–2,950)	3 (2–3)	43.5%	0.55%
Tropical Africa	185 million	148 million (80%)	9,890 (8,250–11,850)	5 (4–6)	35.2%	0.85%
Southeast Asia	165 million	122 million (74%)	15,680 (13,000–18,470)	10 (8–11)	42.4%	1.60%
Indonesia	62.9 million	48.9 million (78%)	6,730 (5,540–7,930)	14 (11–16)	46.4%	0.41%
Malaysia	17.5 million	15.3 million (88%)	2,100 (1,680–2,550)	14 (11–17)	37.3%	1.26%
Vietnam	10.9 million	6.95 million (64%)	2,020 (1,730–2,310)	29 (25–33)	52.0%	0.35%
Democratic Republic of the Congo	51.0 million	42.0 million (82%)	1,840 (1,530–2,190)	4 (4–5)	34.0%	0.31%
Philippines	16.9 million	13.9 million (82%)	1,740 (1,490–2,010)	13 (11–14)	30.9%	0.31%
Nigeria	21.0 million	17.2 million (82%)	1,310 (1,090–1,570)	8 (6–9)	23.1%	0.09%
Mozambique	8.98 million	8.03 million (89%)	1,020 (870–1,230)	13 (11–14)	50.3%	0.47%
Brazil	30.7 million	21.6 million (70%)	990 (890–1,100)	5 (4–5)	38.6%	0.09%
Tanzania	9.35 million	8.03 million (86%)	800 (670–930)	10 (9–12)	107.0%	0.23%
Uganda	11.5 million	9.17 million (80%)	650 (560–770)	7 (6–8)	37.0%	0.27%

The results are shown for populations located in areas of forest loss only, aggregated for the whole tropics, for the three main tropical forest regions and for the ten countries with the greatest deforestation-associated heat-related mortality burdens. All regions are bounded by latitudes of 25° S and 25° N. Populations are only counted in forest loss pixels with valid Δ*T* data and a net forest extent increase of <50%-point. Mortality burdens are shown to the nearest ten for the total mortality and nearest whole number for the mortality rate. The uncertainty ranges are shown in parentheses and are estimated using the 95% CI from the non-accidental mortality rates^40^. Note that deforestation-associated heat-related deaths in a region can exceed the number of total heat-related deaths in that region where surrounding non-deforested areas experience an overall cooling (Methods).

**Extended Data Fig. 1:**
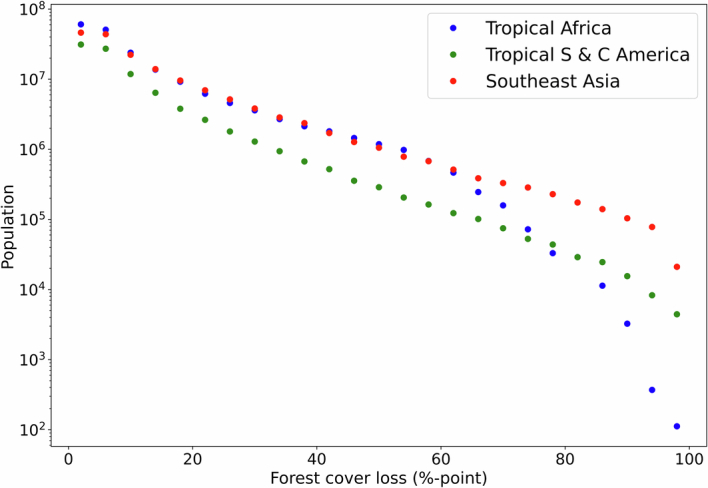
Population as a function of tropical forest loss. Population count in 2020 versus percentage-point forest cover loss between 2001 and 2020. Green points show data for Tropical Central and South America, red points show data for Tropical Africa, and blue points show data for Southeast Asia.

**Extended Data Fig. 2:**
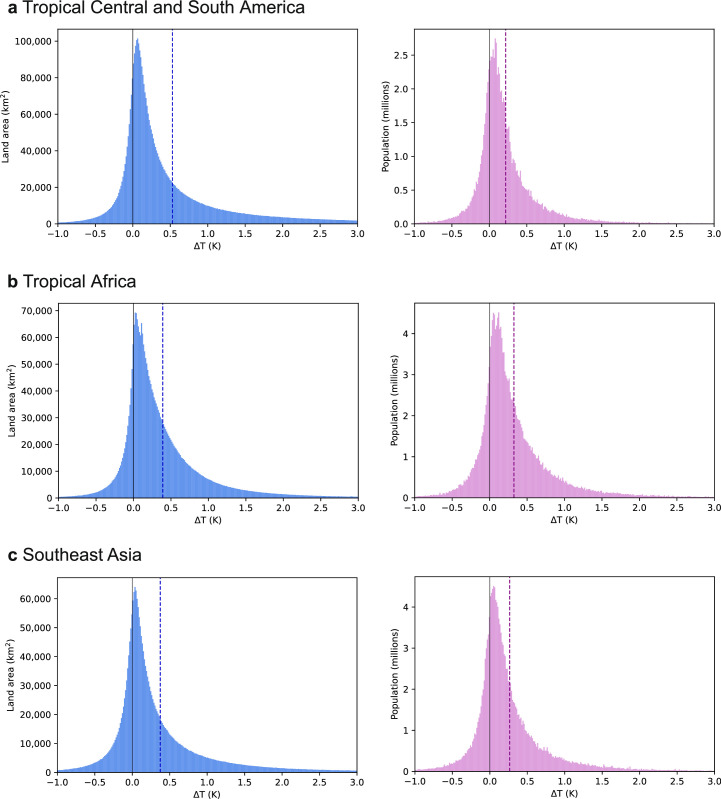
Deforestation-induced ΔT over 2001 to 2020 as a function of area and population. Results are shown for populations in locations of forest loss (see Methods) in **a**. Tropical Central and South America, **b**. Tropical Africa, and **c**. Southeast Asia. Dashed vertical lines show the area- and population-weighted mean deforestation-induced ΔT for each region.

**Extended Data Fig. 3:**
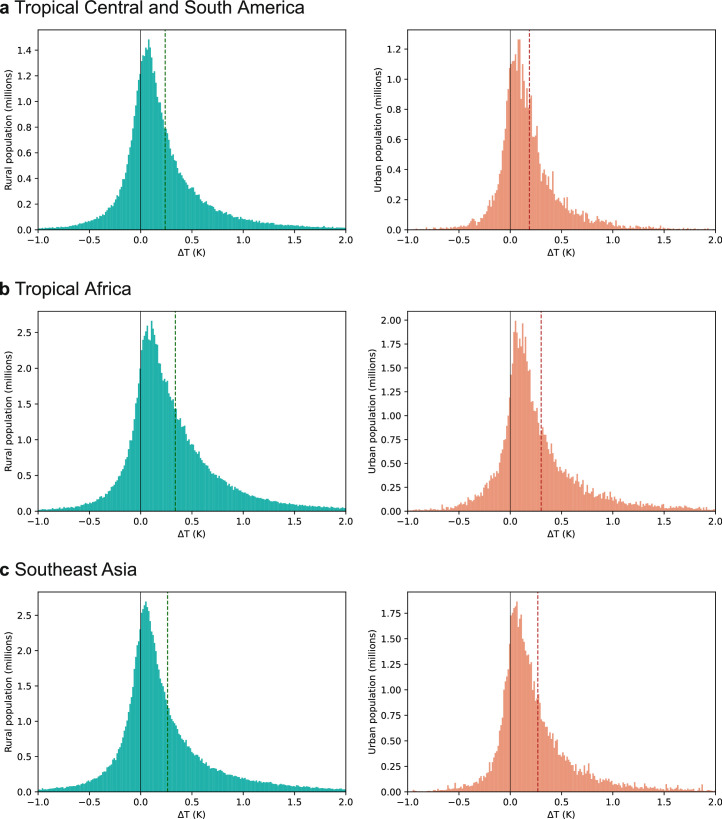
Deforestation-induced ΔT over 2001 to 2020 as a function of rural and urban populations. Results are shown for populations in locations of forest loss in **a**. Tropical Central and South America, **b**. Tropical Africa, and **c**. Southeast Asia. Dashed vertical lines show the population-weighted mean deforestation-induced ΔT for each region.

Over 2001–2020, the populations in areas of tropical forest loss were exposed to a mean Δ*T* of +0.27 °C due to deforestation (Table 1). Regional population-weighted mean Δ*T* is greatest in Tropical Africa (+0.32 °C), where deforestation-induced warming overlaps with more heavily populated areas, and lowest in Tropical Central and South America (+0.22 °C), where deforested areas with strong warming are more sparsely populated (Fig. 1). Across all regions, population-weighted warming due to deforestation is less than area-weighted warming (Table 1) due to lower population densities in rural areas, which typically experience greater forest loss (Extended Data Figs. 1–3).

## Deforestation-associated heat-attributable mortality

**Fig. 3:**
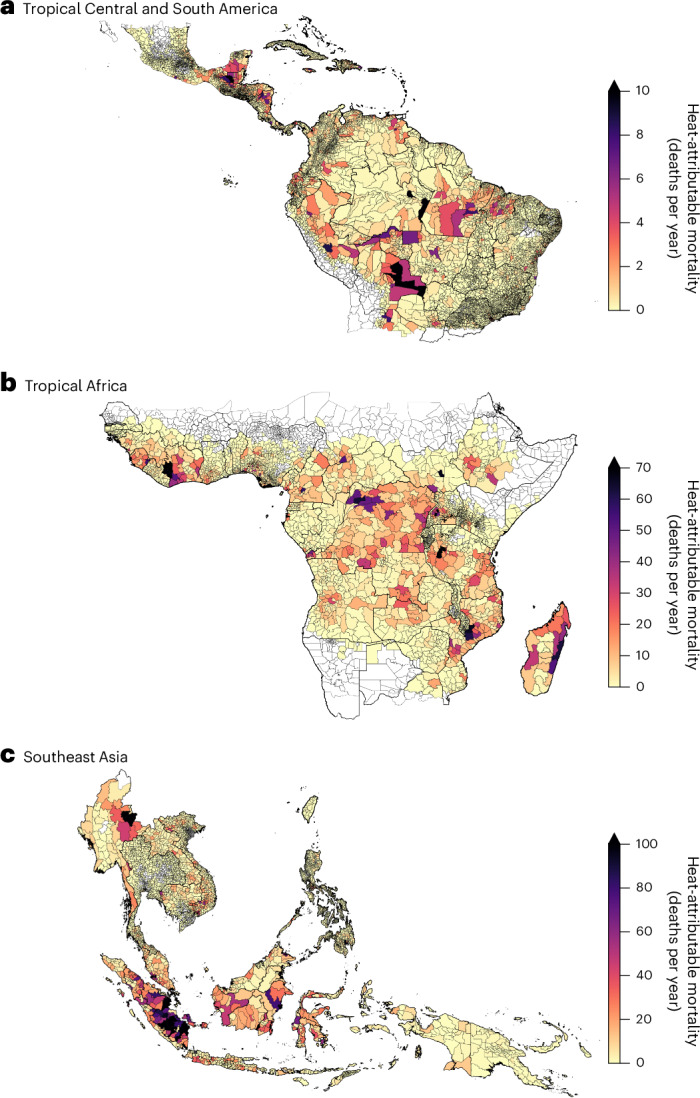
Heat-related non-accidental mortality associated with deforestation-induced warming. **a**–**c**, The maps show regions of Tropical Central and South America (**a**), Tropical Africa (**b**), and Southeast Asia (**c**). The colours show the number of deaths per year (central estimate) for populations located in areas of forest loss, aggregated by second-level administration divisions (boundaries from GADM, https://gadm.org/index.html). Note that the colour scales are different for each region. The administration divisions containing no data points are shown in white.

**Extended Data Fig. 4:**
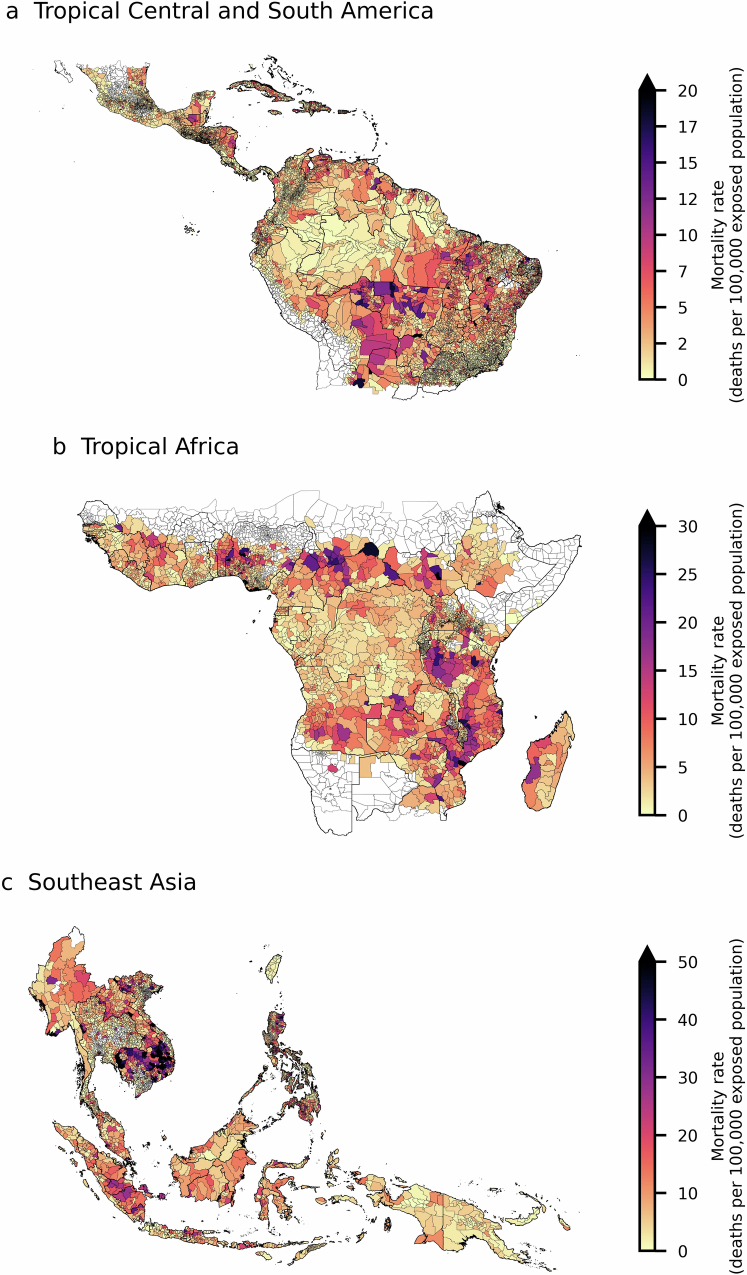
Heat-related non-accidental mortality rate associated with deforestation-induced warming. The maps show regions of Tropical Central and South America (**a**), Tropical Africa (**b**), and Southeast Asia (**c**). Colours show number of deaths per year per 100,000 people located in areas of forest loss (central estimate), aggregated by second-level administration divisions. Boundaries for second-level administration divisions are from GADM (https://gadm.org/index.html).

We estimated the heat-related excess mortality burden associated with deforestation by combining our estimates of deforestation-induced Δ*T* with region-specific heat vulnerability indices^31^ and non-accidental mortality rates (including mortality from all causes apart from external causes)^40^. In general, areas with the greatest mortality burden correspond to regions with moderate to high levels of forest loss and deforestation-induced warming (Fig. 3). However, because the mortality estimates also depend on other factors (population count (Fig. 1c), non-accidental mortality rates and population heat vulnerability (Extended Data Table 2)), the spatial pattern differs from that of the deforestation-induced Δ*T* (Fig. 2). The spatial distribution of the deforestation-associated heat-related mortality rate (that is, deaths per 100,000 people) (Extended Data Fig. 4) is more consistent with deforestation-induced Δ*T* (Fig. 2), demonstrating that the heat-related mortality burden is strongly dependent on the magnitude of the population within areas of forest loss.

Overall, we estimate that warming due to deforestation over 2001–2020 is associated with an additional 28,330 (95% confidence interval (CI): 23,610–33,560) non-accidental deaths per year (Table 2). The estimated deforestation-associated heat-related mortality burden is greatest in Southeast Asia (15,680 (95% CI: 13,000–18,470) annual excess deaths) due to a relatively large exposed population, predominantly in Indonesia, with greater heat vulnerability. In Tropical Africa we estimate 9,890 (95% CI: 8,250–11,850) annual excess deaths associated with deforestation. This is lower than in Southeast Asia, despite greater population exposure to deforestation-induced warming and higher underlying non-accidental mortality rates, because of the lower heat vulnerability that we have assumed for populations in this region (Methods). Although areas of forest loss in Tropical Central and South America experience some of the largest deforestation-induced warming in the tropics, populations in these areas are relatively low, leading to lower population exposure and heat-related mortality (2,520 (95% CI: 2,160–2,950) annual excess deaths) than in the other tropical regions.

## Discussion

Tropical forest regions have warmed substantially over the last two decades due to a combination of climate change and land-use change. We show that over the period 2001–2020, tropical deforestation has caused annual mean land surface warming of 0.7 °C, in line with previous assessments^12–18^. In our analysis, deforestation-induced warming accounts for 64% of the total warming experienced over regions with tropical forest loss (Table 1), demonstrating that deforestation is a major driver of local warming. Deforestation caused 39% of the population-weighted warming experienced over regions with tropical forest loss (Table 1).

Our findings suggest that warming from tropical deforestation impacts large numbers of people and could result in a substantial health burden. We estimate that 345 million people were exposed to local warming from forest loss during 2001–2020. We estimate this warming is associated with an annual heat-related mortality burden of 28,330 (95% CI: 23,610–33,560), equivalent to 39% of the total heat-related mortality (from global climate change and deforestation combined) over locations of forest loss (Table 2). The Global Burden of Disease (GBD) Study^42^ estimated annual excess deaths attributable to high temperature in 2019 to be 14,400 (95% CI: 10,800–18,300) in Latin America and the Caribbean, 50,800 (95% CI: 36,400–66,300) in Sub-Saharan Africa and 41,200 (95% CI: 34,600–49,300) in Southeast Asia. We estimate the total annual heat-related excess deaths (associated with warming from both global climate change and deforestation during 2001–2020) for forest loss regions to be 5,800 (95% CI: 5,000–6,700) in Tropical Central and South America, 28,000 (95% CI: 23,300–33,800) in Tropical Africa and 37,000 (95% CI: 30,700–43,600) in Southeast Asia. Calculating these mortality burdens for the total population, our estimates are comparable with those from the GBD for Latin America and the Caribbean and Sub-Saharan Africa but notably higher for Southeast Asia. However, large differences in methodology, timescale, spatial resolution and temperature datasets complicate direct comparison. Notably, GBD estimates rely on ERA5 (ref. ^41^) near-surface air temperature, which lacks recent and dynamic land cover data (using a monthly climatological vegetation map for the years 2000–2008^43^). Therefore, in regions with sparse weather station coverage, ERA5 does not account for land cover change impacts on local temperature and does not capture increases in temperature with increasing deforestation^44^.

Heat-related mortality from deforestation accounts for 1.1% of non-accidental mortality over regions of tropical forest loss, increasing to 1.6% over Southeast Asia (Table 2). Wolff et al.^10^ estimated that warming from the combination of global climate change and deforestation during 2002–2018 accounted for 7.3–8.5% of all-cause mortality in the Berau Regency in East Kalimantan, Indonesia. In comparison, we estimate warming from global climate change and deforestation during 2001–2020 accounts for 6.7% of all-cause mortality in Berau Regency and 8.0% for populations in locations of forest loss (Extended Data Table 3). We estimate greater population-weighted Δ*T*, due to the inclusion of more years of surface temperature data, but we apply a lower heat vulnerability index to be conservative and to be consistent with the regional average value for Southeast Asia from Lee et al.^31^. We estimate that warming from deforestation during 2001–2020 accounts for 2.0% of all-cause mortality in areas of forest loss in Berau Regency and 34.5% of the total heat-attributable mortality (from global climate change and deforestation combined).

We find that 42% of the tropics-wide heat-related mortality burden associated with deforestation occurs in regions which had greater than 50% forest canopy cover in 2001 compared with 58% in regions of intermediate canopy cover (10–50%) (Extended Data Table 4). Most forest loss occurs in regions that have already been partially deforested such as the Arc of Deforestation (Fig. 1a), which also overlap regions with greater population density (Fig. 1c) and heat-related mortality (Fig. 3). In Africa, forest loss in areas of intermediate canopy cover accounts for 70% of the heat-related mortality burden associated with forest loss. This highlights the impact of forest loss within regions of naturally occurring intermediate canopy cover such as the miombo woodlands of Africa and the Cerrado and Chaco in South America. Our work highlights the importance of sustainable management of these biomes to reduce potential negative health impacts on local populations.

To contextualize the heat-related mortality burden, it is important to consider other health risks associated with tropical deforestation. Smoke pollution from deforestation-related fires can degrade regional air quality^45,46^ leading to adverse health impacts^47,48^. In 2015, peatland fires in Indonesia resulted in exposure of 69 million people in Equatorial Asia to unhealthy air quality conditions^49^. Long-term exposure to particulate air pollution from forest and vegetation fires is estimated to cause ~3,000–16,800 premature deaths annually in South America^50–52^, ~6,000–59,000 in Southeast Asia^49,53,54^ and ~43,000 in Africa^55^. Our estimates of the heat-related mortality burden associated with deforestation-induced warming are comparable with the lower end of estimates of these fire-related mortality estimates.

Tropical deforestation is also linked to increased malaria risk in some contexts^11,56^, with evidence suggesting it is an important driver of childhood malaria risk in poor households^57^. With deforestation-driven malaria incidence estimates only available for the Brazilian Amazon^11^, we compare our results to national-level malaria mortality estimates from the GBD^42^, noting that these may underestimate the burden in deforested areas. In 2019, malaria accounted for 0.04% (95% CI: 0.02–0.07%) of total deaths in Latin America and Caribbean, 7.98% (95% CI: 3.24–15.17%) in Sub-Saharan Africa, and 0.04% (95% CI: 0.01–0.16%) in Southeast Asia^42^. We estimate that heat-related mortality associated with deforestation during 2001–2020 accounts for a smaller proportion of total deaths than malaria in Tropical Africa (0.13%) but greater proportions in Tropical Central and South America (0.08%) and Southeast Asia (0.23%). These results suggest that the deforestation-associated heat-related mortality burden is comparable with other major health burdens linked to tropical deforestation in the Americas and Southeast Asia.

There are no country-specific heat vulnerability indices available for African countries due to lack of data^31^, and so we have used the continental-average heat vulnerability index for South America. Lee et al.^31^ report that the heat vulnerability index shows a large negative dependence on the total health expenditure per capita and a large positive dependence on proportions of obese and elderly populations. A comparison of the key factors that affect heat vulnerability^31^ for Sub-Saharan Africa and other regions and countries (Extended Data Table 5) suggests that using the South American heat vulnerability index for African countries may be conservative. Increasing the heat vulnerability index used for African countries from the South American index (2.34%p °C^−1^) to the Southeast Asian index (5.29%p °C^−1^) more than doubles the deforestation-associated heat-attributable mortality burden estimate for Tropical Africa. Our analysis highlights the critical need for increased climate and health data to improve understanding of heat-health relationships in tropical African countries and other understudied tropical regions.

Our study focused on the local biophysical warming impacts of deforestation (that is, warming within the same 1 km^2^ pixel as the forest loss). Butt et al.^21^ demonstrated that deforestation of the Amazon caused strong warming at distances up to 100 km away from the forest loss. We do not include potential health effects of associated regional temperature changes on populations located outside deforested regions, suggesting we may have underestimated the impacts on human health. Tropical deforestation also releases CO_2_ that contributes to global warming. During 2014–2023, deforestation released 1.7 GtC yr^−1^, accounting for 15% of total anthropogenic CO_2_ emissions^58^. The global warming from release of CO_2_ is not included in our study, meaning that our estimates of heat-related mortality associated with deforestation are likely to be conservative. We estimate that non-deforested regions of the tropics have warmed by 0.2 °C over 2001–2020 (Table 1). Based on the contribution to global emissions, CO_2_ from tropical deforestation may have accounted for 0.03 °C (15%) of this warming, which is substantially less than the biophysical warming (0.45 °C) in regions of tropical deforestation. Nevertheless, the warming from CO_2_ emissions acts globally and the contribution of tropical deforestation to the global burden of heat-related mortality^29^ may be substantial. This highlights the strong potential for reducing tropical deforestation to contribute to reduced warming at both local and global scales.

Future global climate change is projected to increase heat-related mortality in the tropics^31,59^ and severely impact outdoor worker wellbeing and health^60^. Tropical deforestation and its associated local warming are likely to amplify these impacts. Climate model simulations indicate that cropland expansion into tropical forests could elevate local near-surface air temperature and increase heat stress in low-latitude regions^61^. In deforested areas of the tropics, future global climate change is projected to decrease safe outdoor working hours for millions of people^12^. In the Amazon region, scenarios of future climate warming and deforestation project substantial increases in heat stress and reductions in work capacity^62^. Furthermore, compound climate extremes including drought-heatwave episodes, which may be associated with enhanced fire activity and heat-related mortality in Brazil^63^, are increasing in a warming world^64^. In 2023 and 2024, critical episodes of prolonged drought occurred in the Brazilian Amazon, illustrating how climate change intensifies health threats through direct impacts, such as heatwaves and water scarcity, and indirect impacts, such as air pollution from fires.

Lower-income populations in the tropics are already experiencing a greater increase in the frequency of extreme temperatures compared with higher income populations^65^ and are projected to be disproportionately impacted under future global warming^66^. Tropical nations have particularly high heat vulnerability indices, suggesting their populations may be at higher mortality risk due to climate change^31^. This higher heat vulnerability is linked to lower per-capita health expenditure^31^, reflecting persistent socioeconomic disparities in healthcare access^67^. Vulnerable populations, particularly traditional and indigenous communities, often live near deforested areas and face limited access to resources and infrastructure needed to cope with the combination of rising temperatures and environmental changes caused by deforestation and climate change^62^. Our findings suggest that for populations in areas of tropical forest loss, deforestation-driven warming may exacerbate the heat-related health effects of global climate change, with critical implications for outdoor labour^10,12^. Furthermore, these populations may also suffer from disproportionate exposure to other deforestation-related health risks, such as malaria^57^ and fire-sourced air pollution^54^.

Our results underscore the role of deforestation in intensifying local warming within the broader context of climate change. Conservation of tropical forests can help mitigate local warming and strengthen the adaptive capacity of vulnerable populations. Future work should explore how protected areas function as buffers against temperature extremes, particularly for surrounding populations. Overall, our findings highlight the urgent need for targeted policy interventions to reduce tropical forest loss, alongside improved adaptation strategies and access to healthcare, to protect vulnerable populations in the tropics from the health risks associated with deforestation.

## Methods

### Temperature datasets

As in previous work^17,21^, we used land surface temperature (LST) data from NASA MODIS, specifically the MOD11A2 8-d LST data (here using the latest version 6.1)^38^ at 0.01° × 0.01° spatial resolution. We excluded data where the estimated emissivity error was greater than 0.02 and where the LST error was greater than 1 K, following Li et al.^13^. We used MODIS data from the Terra satellite (10:30 local overpass time) because of its longer sampling period compared with the Aqua satellite (February 2000 onwards versus July 2002 onwards) (13:30 overpass time) and due to lower cloud cover in the morning.

We first aggregated the 8-day LST data by month ignoring any 8-day period where data were missing due to clouds or because of the quality screening process. We then calculated 3-year means for two periods at the start (2001–2003) and end (2018–2020) of the study period. Using 3-year averages reduces the influences of climate variability. The LST dataset was regridded using bilinear interpolation (using Python package xESMF^68^) to match the finer resolution of the population data grid (30 arc-seconds). The Δ*T* in multiannual mean LST was then calculated by subtracting the 2001–2003 mean LST from the 2018–2020 mean LST. Our final Δ*T* dataset comprised over 64 million pixels (~1 km^2^ in extent) over the tropics (25° S to 25° N).

To assess the impacts of using morning LST data in place of daily mean air temperature data (as used by Lee et al.^31^ to derive the heat vulnerability indices), we used 2-m temperature (t2m) and LST data from the ERA5 (ref. ^41^) at 0.25° × 0.25° spatial resolution. We downloaded the hourly Analysis-Ready, Cloud Optimized (ARCO) ERA5 data from the Google Cloud Public Dataset Program^69^ for the years 2001–2003 and 2018–2020. First, we calculated daily mean t2m and extracted the ERA5 LST data at 10:00–11:00 local time. Second, we aggregated both variables by month and calculated 6-year means, regridding to match the population data grid (30 arc-seconds). Third, we calculated the relationship between ERA5 LST and ERA5 t2m over tropical forest pixels (using 2001 forest cover) of the main tropical forest regions (Extended Data Table 1).

### Forest cover datasets

Forest cover data were taken from the Global Forest Change (GFC) V1.9 dataset^1^ at 30 m × 30 m spatial resolution. Annual forest cover for the period 2000–2020 was calculated by taking tree cover in the year 2000, defined as canopy closure for all vegetation taller than 5 m in height, and subjecting it to annual forest loss, defined as a disturbance from a forest to non-forest state. Forest cover was calculated at the native 30 m × 30 m spatial resolution and then converted to match the resolution of the population data by first calculating the forest cover fraction in each larger pixel (0.008° × 0.008°) and then converting to a 30 arc-second resolution using area-weighted regridding (using Python package Iris^70^). The percentage-point change in forest cover between 2001 and 2020 was calculated as the difference between the annual forest cover fractions (in per cent) in 2001 and 2020.

Forest cover gain can lead to cooling that can offset some of the warming due to forest loss^71^. To identify and exclude areas where forest regrowth has occurred, we used forest extent data at 30 m × 30 m spatial resolution from the Global Land Analysis and Discovery (GLAD) Global Land Cover and Land Use Change dataset^72,73^, available for the years 2000 and 2020. In the forest extent dataset, forest presence is indicated for pixels with 5 m or greater forest height. We converted this dataset to match the resolution of the population data using the same method as for the GFC data and calculated the percentage-point difference in forest extent between 2000 and 2020. We then removed any 30-arc-second pixels with greater than a 50%-point net increase in forest extent between 2000 and 2020 from the Δ*T* data.

### Elevation dataset

We used elevation data from the Global Multi-resolution Terrain Elevation Data (GMTED2010)^74^ at 7.5-arc-second spatial resolution, which was regridded using bilinear interpolation to match the resolution of the population data.

### Population and mortality datasets

Spatially explicit population data for the year 2020 was taken from LandScan^75,39^ at 30-arc-second spatial resolution representing an average population over 24 h (Fig. 1c). We used LandScan data for 2001 to test the sensitivity of our results to changing population density and distribution (‘Methodological limitations and justification’ section). To explore exposure of rural and urban populations, we used the Settlement Model Layers data from the Global Human Settlement Layer (GHSL) 2023 Data Package^76,77^.

Annual all-cause mortality and non-accidental mortality rates for all ages for the year 2019 were taken from the Global Burden of Disease Study (GBD)^40^. Non-accidental mortality (or non-external mortality) includes mortality from all causes except external causes such as accidents, suicides and homicides. We used data from 2019 instead of 2020 to exclude possible effects of coronavirus disease 2019 on the mortality rates. We used the highest resolution data available from the GBD^40^: province/state level data for Brazil and Indonesia and national-level data for all other countries in the tropics. We used GBD non-accidental mortality rates from 2001 to test the sensitivity of our results to changes in the annual mortality rates (‘Methodological limitations and justification’ section).

To estimate total annual all-cause and non-accidental mortality burdens at the pixel level, we multiplied the GBD mortality rates by the gridded total population data (pop)

1 $${\mathrm{nonacc}}{\_}{\mathrm{mort}}_{i}={\mathrm{pop}}_{i}\times{\mathrm{nonacc}}_{\_}{\mathrm{mortrate}}_{\mathrm{country}},$$

where nonacc_mortrate_country_ is the national (or subnational) non-accidental mortality rate and nonacc_mort_*i*_ is the total annual non-accidental mortality burden per pixel (*i*).

### Heat vulnerability indices

To estimate the heat-related excess mortality burden, we used country- or continent-specific heat vulnerability indices from Lee et al.^31^ following Wolff et al.^10^. The heat vulnerability index is the percentage-point increase of regional excess mortality per 1 °C increase of regional temperature (%p °C^−1^). Lee et al.^31^ first estimated location-specific temperature-mortality relationships for 459 locations in 28 countries using daily mean temperature, then estimated the projected excess mortality attributable to temperature change over 2010–2099 using a calibrated multimodel mean temperature time-series under different scenarios. The heat vulnerability indices were obtained for each location by applying linear regressions to the projection data^31^.

The heat vulnerability indices were estimated by Lee et al.^31^ for 10 continental regions and 28 countries; those used in this study are shown in Extended Data Table 2. Where country-specific indices were not available, we applied a continent-specific value. Following Lee et al.^31^, for countries located in Central and South Asia, we applied the heat vulnerability index from Iran; for countries located in Central America, we applied the index from Mexico. Lee et al.^31^ were unable to derive indices for African countries due to a lack of data; therefore, we have applied the lowest continental-average index (from South America) for the whole of the Tropical Africa.

### Estimating deforestation-induced temperature change

We estimated the local Δ*T* due to forest loss between 2001 and 2020 at the 30-arc-second (~1 km^2^) pixel level across the tropics (25° S to 25° N). To remove the influence of global climate change over the study period on Δ*T*, we used a moving-window nearest-neighbour approach^7,17^.

In our analysis we used all 30-arc-second pixels within the tropics (25° S to 25° N) with 10% or greater forest cover fraction in 2001. Pixels with less than 0.5%-point forest cover loss between 2001 and 2020 were classified as ‘non-deforested’ pixels. Pixels with 2%-point or greater forest cover loss between 2001 and 2020 were classified as ‘deforested’ pixels. Our final Δ*T* dataset comprised ~16.8 million non-deforested pixels and ~11.3 million deforested pixels across the tropics (25° S to 25° N).

For each deforested pixel, we selected all surrounding pixels that maintained forest cover (‘non-deforested’ pixels) within a circle of 0.25° radius (~27 km at the equator) and within an elevation of ±50 m. If no surrounding non-deforested pixels were available within the 0.25° radius circle (3% of pixels), the radius of the circle was extended to 0.50°, with the same elevation constraints of within ±50 m. We used 0.25° and 0.5° radius circles similar to those used in previous work (for example, refs. ^13,14,16^). These previous studies—and ours—assume the background climate to be uniform over this distance. We calculated the mean Δ*T* over all selected non-deforested (‘control’) pixels and subtracted this from the Δ*T* of the central deforested pixel to get the deforestation-induced Δ*T*. A total of 0.58% of deforested pixels across the tropics were without surrounding non-deforested pixels within the specified spatial circle and elevation range; these pixels were retained in the data array but stored with a missing data indicator. We then divided the resulting deforestation-induced Δ*T* data array by the percentage-point change in forest cover to calculate the change in temperature per percentage-point forest cover loss.

At low levels of forest cover loss (<10%-point), the variability in Δ*T* per percentage-point forest cover loss exceeds the range in values found at greater levels of forest cover loss. To reduce this variability, we used a moving average smoothing approach. For each deforested pixel, we calculated the mean Δ*T* per percentage-point forest cover loss over all surrounding deforested pixels within a 0.10° rolling circle, excluding any pixel with less than 5%-point forest cover loss or with a *z*-score greater than 3 from the mean. For deforested pixels without surrounding deforested pixels with ≥5%-point forest cover loss within the 0.10° spatial circle (0.4% of all deforested pixels across the tropics), we used a regional mean value for Δ*T* per percentage-point forest cover loss. Finally, we multiplied the ‘smoothed’ pixel-level Δ*T-*per-percentage-point-forest-cover-loss values by the pixel-level percentage-point change in forest cover to obtain a more robust estimate of the pixel-level deforestation-induced Δ*T*.

### Estimating deforestation-associated heat-related mortality

To estimate heat-related excess mortality, we followed the approach of Wolff et al.^10^, using the relationships between heat-attributable excess mortality and temperature estimated by Lee et al.^31^. Wolff et al.^10^ estimated heat-related mortality due to global climate change and deforestation combined. In this study, we estimated the heat-related mortality due specifically to local deforestation-induced warming.

First, we calculated the pixel-level deforestation-induced Δ*T* over 2001–2020 in all deforested pixels across the tropics (as described above). Second, to estimate the percentage-point increase in heat-attributable excess mortality (%pincrease_mort) per deforested pixel (*i*), we multiplied the country/continent-specific heat vulnerability indices (HVI) by the pixel-level Δ*T* in all deforested pixels with a positive temperature change (79% of all deforested pixels across the tropics) (Δ*T*defor)

2 $${ \% {{\mathrm{pincrease}}}{\rm{\_}}{{\mathrm{mort}}}}_{i}={\Delta {T{\mathrm{defor}}}}_{i}\times {{{\mathrm{HVI}}}}_{{{\mathrm{country}}}}.$$

The mortality impact of the 2001–2020 deforestation-induced warming will already be included in the non-accidental mortality burden data from 2019 (‘Population and mortality datasets’ section). Thus, following Wolff et al.^10^, we next calculated the corrected counterfactual mortality burden (CF_mort) (that is, without the excess mortality associated with the deforestation-induced Δ*T* that would already be included in the total non-accidental mortality burden) per deforested pixel (*i*)

3 $${{{\mathrm{CF}}}{{\_}}{{\mathrm{mort}}}}_{i}=\frac{{{{\mathrm{nonacc}}}{{\_}}{{\mathrm{mort}}}}_{i}}{\left(1+{ \% {{\mathrm{pincrease}}}{{\_}}{{\mathrm{mort}}}}_{i}\right)}.$$

Lastly, we calculated the mortality attributable to warming from deforestation for each deforested pixel, as the difference between the counterfactual non-accidental mortality and total non-accidental mortality

4 $${{{\mathrm{heat}}}{\rm{\_}}{{\mathrm{mort}}}}_{i}={{{\mathrm{nonacc}}}{\rm{\_}}{{\mathrm{mort}}}}_{i}-{{{\mathrm{CF}}}{\rm{\_}}{{\mathrm{mort}}}}_{i}.$$

The results are estimated using annual non-accidental mortality rates and therefore represent an annual excess mortality burden associated with exposure to a ~20-year temperature change. Following Wolff et al.^10^, we refer to the heat-health impact results as an annual excess heat-attributable mortality burden. The uncertainty range in the heat-attributable mortality estimates was calculated based on the uncertainty range (the 95% CI) of the GBD all-cause/non-accidental mortality rates^40^.

For comparison, we also estimated the total heat-related mortality attributable to warming over 2001–2020 from global climate change and deforestation combined. This was done using the same method as above but replacing the deforestation-induced Δ*T* with the total Δ*T*. We note that where a deforested pixel experiences warming but surrounding non-deforested (‘control’) pixels experience an overall cooling, the deforestation-induced Δ*T* will be greater than the total Δ*T*, and the number of deforestation-associated heat-related deaths will exceed the number of total heat-related deaths in that pixel.

### Methodological limitations and justification

We defined forested locations in the tropics as pixels with 10% forest canopy cover of vegetation taller than 5 m in height, which is consistent with the definition from the Food and Agriculture Organization of the United Nations^78^. We defined deforestation to be a net loss in forest cover of 2%-points or greater between 2001 and 2020. We accounted for dynamics of forest cover change within the given time period, by using the GLAD forest extent dataset to remove pixels with a >50%-point net increase in forest extent between 2000 and 2020. We tested the sensitivity to these assumptions. Defining deforestation as net forest loss greater than 1%-point, increased the number of data pixels and population included in our analysis, increasing our estimated mortality burden by 9% (Extended Data Table 4), but remains within our cited uncertainty range. Applying a lower threshold to the net increase in forest extent (20%-point) reduced the population exposed to deforestation-associated warming by only 1% (Extended Data Table 4).

We analysed the change in forest cover and LST between 2001 and 2020. The years of data included in the analysis were selected to maximize the study period with the available data, while also avoiding strong ENSO years in our 3-year averages of the LST data (2001–2003 and 2018–2020). We note that our analysis method attempts to remove the impact of climate variability through comparing temperature change over regions of forest loss with nearby regions with no forest loss. For example, during El Nino periods, both regions of forest loss and regions of no forest loss will experience a warming due to the impacts of El Nino. Our analysis pulls out the impact of forest loss during the El Nino by subtracting the temperature of surrounding forested pixels. We tested the sensitivity to the selected years of LST data by recalculating for a different 3-year period (from 2003–2005 to 2018–2020), 5-year periods (from 2001–2005 to 2016–2020) and for single years (2001–2019 and 2001–2020). Estimated deforestation-associated heat-related mortality burdens for these different time periods lie within our 95% CIs (varying by −13% to +15%) (Extended Data Table 4), showing that our method is robust to these assumptions. Averaging the forest loss data to match the same years as the LST data (from 2001–2003 to 2018–2020) reduces the heat-related mortality burden associated with deforestation by only 2% (Extended Data Table 4).

We used LST data to estimate human exposure and heat-related health impacts of deforestation-induced warming rather than near-surface air temperature, because air temperature data are not available at sufficiently high spatial resolution and coverage for the study time period. The use of LST data for exploring fine-scale temperature changes under tropical forest loss has been discussed and justified in previous studies^10,12,17^. For tropical forest regions, there is a particularly uneven and sparse distribution of ground-based meteorological stations^79,80^, which means that air temperature measurements from these stations (or datasets that spatially interpolate these measurements) are not appropriate for a tropics-wide assessment of fine-scale deforestation-induced temperature changes. Where meteorological stations exist, previous studies have shown a tight connection between LST and near-surface air temperature^14,81–83^. Estimates of near-surface air temperature from climate reanalyses, such as the ERA5 (ref. ^41^), have been used extensively to estimate human exposure to extreme heat and the associated health impacts^40,84–86^. While the relatively coarse resolution of ERA5 data make it unsuitable for examining fine-scale temperature changes driven by deforestation, it is reported to well capture changes in air temperature where meteorological station density is high^79^. To assess the impacts of using LST data in place of air temperature data, we used the relationship between ERA5 daily mean t2m and ERA5 10:00–11:00 local time LST (Extended Data Table 1) to adjust MODIS LST to daily mean 2-m air temperature. We then recalculated the impacts of forest loss using this adjusted ‘air temperature data’, which reduces the deforestation-associated heat-attributable mortality burden by only 1% (Extended Data Table 4).

We used morning measurements of LST from MODIS-Terra for its greater data availability compared with MODIS-Aqua (an overpass of 13:30 local time). Furthermore, MODIS-Terra tends to sense a cooler land surface than MODIS-Aqua and is therefore more representative of the daily mean temperature data used to derive the heat vulnerability indices by Lee et al.^31^. Using afternoon LST data from MODIS-Aqua (for 2003–2005 mean to 2018–2020 mean) increased the population-weighted warming associated with deforestation to +0.29 °C and increased the estimated deforestation-associated heat-attributable mortality burden to 30,590 (95% CI: 25,520–36,230) (Extended Data Table 4). This mortality burden is 19% greater than with MODIS-Terra LST for the same period and within our stated 95% CI.

In this work, we have not assessed the potential impact of deforestation on humidity. In situ measurements^87,88^ and reanalysis data^89^ show a general reduction in humidity in cleared areas of the tropics compared with forested areas, although this difference can be seasonally dependent. Results of a modelling study suggest that changes in humidity under simulated cropland expansion into low-latitude forested areas could moderate combined impacts on moist heat and heat stress^61^. However, Masuda et al.^26^ and Parsons et al.^12^ show that despite potential deforestation-induced changes in humidity, humid heat exposure increases substantially in areas of tropical forest loss compared with nearby forested areas, negatively impacting outdoor workers. There is a strong need for field measurements of humidity in tropical forest areas to enable further research into impacts of deforestation on humidity and the associated health impacts

Following Wolff et al.^10^, we have not included an estimation of the change in cold-related mortality under higher temperatures. For tropical countries, the reduction in cold-related mortality under increasing temperatures is small and is outweighed by the associated increase in heat-related mortality^31^.

The heat vulnerability indices were developed by Lee et al.^31^ using either non-accidental or all-cause mortality rates (Extended Data Table 2), depending on the data available for each country. For simplicity we report results estimated using non-accidental mortality rates. Using all-cause mortality rates in all locations increases the total deforestation-associated heat-attributable mortality burden across the tropics by ~8% (with a 15% increase in Tropical Central and South America where differences between non-accidental and all-cause mortality rates are greatest).

We report heat-related mortality estimates that are relevant for a near-present-day population, using population count and spatial distribution for 2020 and non-accidental (or all-cause) mortality rates for 2019. During 2001–2020, population count has increased across the tropics, while changes in non-accidental mortality rates are mixed. Using population count and spatial distribution and non-accidental mortality rates for the start of the study period (2001) reduces the total deforestation-associated heat-attributable mortality burden across the tropics by ~10%.

### Reporting summary

Further information on research design is available in the Nature Portfolio Reporting Summary linked to this article.

## Online content

Any methods, additional references, Nature Portfolio reporting summaries, source data, extended data, supplementary information, acknowledgements, peer review information; details of author contributions and competing interests; and statements of data and code availability are available at 10.1038/s41558-025-02411-0.

## Supplementary information

Reporting summary

## Data Availability

The datasets used in the analyses for this study are freely available to download from the following locations: MODIS land surface temperature data at https://www.earthdata.nasa.gov/; European Centre for Medium-Range Weather Forecasts Reanalysis v5 (ERA5) Analysis-Ready, Cloud Optimized (ARCO) dataset on GitHub at https://github.com/google-research/arco-era5; Global Forest Change (GFC) dataset at https://storage.googleapis.com/earthenginepartners-hansen/GFC-2023-v1.11/download.html; Global Land Analysis and Discovery (GLAD) Global Land Cover and Land Use Change dataset at https://glad.umd.edu/dataset/GLCLUC2020; Global Multi-resolution Terrain Elevation Data (GMTED2010) at https://earthexplorer.usgs.gov/; LandScan population data at https://landscan.ornl.gov/; Global Human Settlement Layer dataset at https://human-settlement.emergency.copernicus.eu/ghs_smod2023.php; GBD Study cause-specific mortality rates at https://gbd2019.healthdata.org/gbd-results/ or https://vizhub.healthdata.org/gbd-results/; and digital geospatial data for plotting administrative boundaries from Database of Global Administrative Boundaries (GADM) at https://gadm.org/index.html. The processed data files produced and used in the analyses of this study are available through Code Ocean^90^.

## Extended data

**Extended Data Table 1:** Comparison of ERA5 2-m air temperature (t2m) with MODIS land surface temperature (LST) and ERA5 LST

Region (25S-25N)	ERA5_t2m_daily / MODIS_LST (2001 tropical forest pixels)	ERA5_t2m_daily / ERA5_LST_daily	ERA5_t2m_daily / ERA5_LST_10–11amLT	ERA5_t2m_daily / ERA5_LST_10-11amLT (2001 tropical forest pixels)
Tropical C & S America	0.9835	0.9989	0.9854	0.9887
Tropical Africa	0.9652	0.9960	0.9755	0.9835
Tropical Central Asia	0.9655	0.9974	0.9801	0.9837
Southeast Asia	0.9757	0.9969	0.9840	0.9906

Values shown are ratios between daily-mean ERA5 t2m and either MODIS-Terra LST (at ~10:30am local time (LT)), daily mean ERA5 LST, or hourly ERA5 LST at 10:00–11:00 am LT. Ratios are calculated at the ~1-km-pixel-level between 6-year (2001–2003 and 2018–2020) regional means. Ratios for MODIS LST at ERA5 LST at 10:00–11:00 am LT are calculated over tropical forest pixels only (using 2001 forest cover fraction).

**Extended Data Table 2:** Heat vulnerability indices estimated by Lee et al.^31^

Country / continent	Data period	Number of locations	Heat Vulnerability (%p °C^−1^)	Mortality causes
Mexico	1998–2014	10	1.83	All
Central America		10	1.83	−
Argentina	2005–2015	3	2.07	Non-external
Brazil	1997–2011	18	2.57	Non-external
Chile	2004–2014	2	2.49	All
Colombia	1998–2013	5	1.62	[Not provided]
South America		28	2.34	−
Iran	2004–2013	1	2.93	Non-external
Central Asia		1	2.93	−
Philippines	2006–2010	4	9.15	All
Taiwan	1994–2007	3	2.83	Non-external
Thailand	1999–2008	56	4.39	Non-external
Vietnam	2009–2013	2	11.82	Non-external
Southeast Asia		65	5.29	−
Australia	1988–2009	3	1.02	Non-external
China	1996–2008	16	1.46	Non-external

Data used to estimate the indices were taken from the database collected through the Multi-City Multi-Country (MCC) Collaborative Research Network. Relevant information on the data is included in the table; additional information on the MCC dataset can be found in Gasparrini et al.^96^. Causes of mortality considered depend on available data (either all-cause or non-external/non-accidental mortality).

**Extended Data Table 3:** Comparison of results and methods from this study to Wolff et al.^10^

Reference	Wolff et al.^10^	This study (sensitivity experiments)						This study (main results)
LST data	Afternoon LST (MODIS-Aqua)	Morning LST (MODIS-Terra)						
Population data & year (Population of Berau)	Berau Census 2018 & LandScan 2017 (232,528)	LandScan 2020 (206,331)						
LST time period	2002 to 2018	2001–2003 to 2017–2019		2001–2003 to 2018–2020				
Forest loss threshold in "kept forest" pixel	0%p	0%p	<0.5%p	0%p	<0.5%p	<0.5%p	<0.5%p	<0.5%p
Mean ΔT in forest loss pixels (°C)	1.03	0.80	0.80	1.05	1.05	1.05	1.05	1.05
Mean ΔT in kept forest pixels (°C)	0.08	0.10	0.18	0.36	0.43	0.43	0.43	0.43
Δpop-wgtd T (°C)	0.86	1.61	1.61	1.36	1.36	1.36	1.36	1.36
Heat vulnerability index (%p °C^−1^)	9.15	9.15	9.15	9.15	9.15	9.15	5.29	5.29
Mortality cause (deaths per 100,000 in Berau)	All-cause (596)	All-cause (532)	All-cause (532)	All-cause (532)	All-cause (532)	Non-accidental (495)	All-cause (532)	Non-accidental (495)
Increase in heat-related deaths in Berau (heat-attributable increase in total mortality)	101 (7.3%)	141 (12.9%)	141 (12.9%)	121 (11.0%)	121 (11.0%)	113 (11.0%)	73 (6.7%)	68 (6.7%)
Pop-wgtd mean ΔT^a^ (°C)		1.53	1.53	1.68	1.68	1.68	1.68	1.68
Increase in heat-related deaths^a^ (heat-attributable increase in total mortality^a^)		93 (11.9%)	93 (11.9%)	101 (12.9%)	101 (12.9%)	94 (12.9%)	63 (8.0%)	58 (8.0%)
**Deforestation-associated values:**								
Pop-wgtd mean ΔT^a^ (°C)		0.60	0.54	0.58	0.55	0.55	0.55	0.55
Increase in heat-related deaths^a^ (heat-attributable increase in total mortality^a^)		39 (3.6%)	35 (3.2%)	38 (3.5%)	36 (3.3%)	34 (3.3%)	22 (2.0%)	20 (2.0%)
Contribution to total heat-attributable mortality^a^		42.3%	38.1%	37.8%	35.7%	35.7%	34.5%	34.5%

Sensitivity tests varied methodological assumptions to more closely match those of Wolff et al.^10^, including i) the time period of Land Surface Temperature (LST) data used, ii) the ‘kept forest’ (or ‘non-deforested’) pixel definition applied, iii) the heat vulnerability index used, and iv) the mortality cause considered. Differences in some assumptions remain, for example, in the forest cover definition ( >50% in 2000 in Wolff et al.^10^ and ≥10% in 2001 in this study) and ‘deforested pixel’ definition ( >0%p forest loss in Wolff et al.^10^ and ≥2%p forest loss in this study). The methods and results in the final column are those presented in the main text of this study.

^a^In forest loss pixels only.

**Extended Data Table 4:** Comparison of deforestation-associated heat-related mortality results for different time-periods and forest-cover-related thresholds

ΔT data period	T data	Forest cover fraction (in 2001)	Net increase in forest extent threshold	Forest cover loss in ‘deforested’ pixels	Pop in locations of forest loss (millions)	Pop exposed to deforestation-induced warming (millions)	Annual deforestation-associated heat-related mortality	Annual deforestation-associated heat-related mortality rate (deaths / 100,000 people)
2001–2003 to 2018–2020	MODIS-Terra LST	≥10%	>50%p	≥2%p	452	345 (76%)	28,330 (23,610–33,560)	6 (5–7)
2001–2003 to 2018–2020	MODIS-Terra LST	≥10%	>50%p	**≥10%p**	147	119 (84%)	13,830 (8,480–16,400)	9 (6–11)
2001–2003 to 2018–2020	MODIS-Terra LST	≥10%	>50%p	**≥1%p**	602	456 (76%)	30,790 (15,670–36,460)	5 (4–6)
2001–2003 to 2018–2020	MODIS-Terra LST	**≥50%**	>50%p	≥2%p	169	128 (76%)	11,840 (9,820–14,030)	7 (6–8)
2001–2003 to 2018–2020	MODIS-Terra LST	≥10%	**>20%p**	≥2%p	448	341 (76%)	27,570 (22,980–32,650)	6 (5–7)
**2001 to 2019**	MODIS-Terra LST	≥10%	>50%p	≥2%p	446	319 (71%)	32,200 (26,830–38,110)	7 (6–9)
**2001 to 2020**	MODIS-Terra LST	≥10%	>50%p	≥2%p	445	322 (72%)	32,600 (27,170–38,620)	7 (6–9)
**2001–2003 to 2018–2020 (LST & forest cover)**	MODIS-Terra LST	≥10%	>50%p	≥2%p	429	331 (76%)	27,670 (23,050–32,770)	6 (5–8)
**2001–2005 to 2016–2020**	MODIS-Terra LST	≥10%	>50%p	≥2%p	453	344 (76%)	24,720 (20,610–29,270)	7 (6–8)
**2003–2005 to 2018–2020**	MODIS-Terra LST	≥10%	>50%p	≥2%p	452	328 (73%)	25,670 (21,390–30,410)	6 (5–7)
**2003–2005 to 2018–2020**	**MODIS-Aqua LST**	≥10%	>50%p	≥2%p	445	323 (73%)	30,590 (25,520–36,230)	7 (6–8)
2001–2003 to 2018–2020	**MODIS-Terra LST scaled by ERA5**	≥10%	>50%p	≥2%p	452	345 (76%)	28,000 (23,340–33,170)	6 (5–7)

Changing time-periods and threshold assumptions can change the number of pixels classed as tropical forest and ‘deforested’ in our analysis and therefore the magnitude of the population in pixels of forest loss. Note in this work, populations in forest loss pixels are only counted where the net forest extent increase is below the selected threshold and land surface temperature (LST) data pixels are valid.

**Extended Data Table 5:** Data on key factors affecting the heat vulnerability indices^31^

Region/country	Current health expenditure per capita (current US$) (2020)^97^	Prevalence of overweight (% of adults) (2016)^98^	Population ages 65 and above (% of total population) (2020)^99^
Sub-Saharan Africa	74.6	28.9	3.1
Latin America & Caribbean	598.0	59.4	8.8
Latin America & Caribbean (excluding high income)	584.6	59.1	8.6
East Asia & Pacific	792.8	31.2	12.1
East Asia & Pacific (excluding high income)	448.0	31.0	10.9
Argentina	895.1	62.7	11.7
Australia	5958.8	64.5	16.2
Brazil	705.0	56.5	9.3
Chile	1280.6	63.1	12.4
China	583.4	32.3	12.6
Colombia	462.2	59.0	8.5
Iran, Islamic Rep.	338.2	61.6	7.1
Mexico	538.5	64.9	8.0
Philippines	166.0	**27.6**	**5.2**
Thailand	305.1	32.6	13.9
Viet Nam	**154.2**	18.3	8.4

Values are shown for three broad tropical regions and for the individual countries with heat vulnerability indices available. Values highlighted in bold are those closest to the values for Sub-Saharan Africa.

## Code availability

The data were analysed using open-source Python packages (including xESMF^68^, Iris^70^, xarray^91^, NumPy^92^, Matplotlib^93^, Pandas^94^ and Cartopy^95^) that are freely available online. The Python code used to produce the central results of this study is available as a Compute Capsule through Code Ocean^90^. Any other Python code used is available upon request from the corresponding author.
