# Supplementary material for: Tropical deforestation is associated with considerable heat-related mortality
Source: Nat Clim Chang. 2025 Aug 27;15(9):992–9. doi: 10.1038/s41558-025-02411-0 (PMC12417208; doi:10.1038/s41558-025-02411-0)
Supplement: Supplementary file 1 — Reporting summary [file 41558_2025_2411_MOESM1_ESM.pdf]

nature portfolio

Corresponding author(s): Carly Reddington

Last updated by author(s): Jul 16, 2025

Reporting Summary

Nature Portfolio wishes to improve the reproducibility of the work that we publish. This form provides structure for consistency and transparency in reporting. For further information on Nature Portfolio policies, see our Editorial Policies and the Editorial Policy Checklist.

Statistics

For all statistical analyses, confirm that the following items are present in the figure legend, table legend, main text, or Methods section.

n/a	Confirmed	
☐	☒	The exact sample size (*n*) for each experimental group/condition, given as a discrete number and unit of measurement
☒	☐	A statement on whether measurements were taken from distinct samples or whether the same sample was measured repeatedly
☒	☐	The statistical test(s) used AND whether they are one- or two-sided<br>*Only common tests should be described solely by name; describe more complex techniques in the Methods section.*
☒	☐	A description of all covariates tested
☒	☐	A description of any assumptions or corrections, such as tests of normality and adjustment for multiple comparisons
☐	☒	A full description of the statistical parameters including central tendency (e.g. means) or other basic estimates (e.g. regression coefficient) AND variation (e.g. standard deviation) or associated estimates of uncertainty (e.g. confidence intervals)
☒	☐	For null hypothesis testing, the test statistic (e.g. *F*, *t*, *r*) with confidence intervals, effect sizes, degrees of freedom and *P* value noted<br>*Give P values as exact values whenever suitable.*
☒	☐	For Bayesian analysis, information on the choice of priors and Markov chain Monte Carlo settings
☒	☐	For hierarchical and complex designs, identification of the appropriate level for tests and full reporting of outcomes
☒	☐	Estimates of effect sizes (e.g. Cohen's *d*, Pearson's *r*), indicating how they were calculated

Our web collection on statistics for biologists contains articles on many of the points above.

Software and code

Policy information about availability of computer code

Data collection	No data collection was performed for this study.
Data analysis	Used open-source Python v3.11.6 with the following open-source packages: xESMF v0.8.2, Iris v3.1.0, xarray v2023.11.0, NumPy v1.26.2, Matplotlib v3.9, Pandas v2.1.3, and Cartopy v0.24.1.(Citations for the Python packages used are provided in the manuscript). Python code used to produce the central results of this study will be available as a Compute Capsule through Code Ocean upon acceptance (provisional DOI: 10.24433/CO.9925363.v1; https://codeocean.com/capsule/9925363/tree).

For manuscripts utilizing custom algorithms or software that are central to the research but not yet described in published literature, software must be made available to editors and reviewers. We strongly encourage code deposition in a community repository (e.g. GitHub). See the Nature Portfolio guidelines for submitting code & software for further information.

Data

Policy information about availability of data

All manuscripts must include a data availability statement. This statement should provide the following information, where applicable:

- Accession codes, unique identifiers, or web links for publicly available datasets
- A description of any restrictions on data availability
- For clinical datasets or third party data, please ensure that the statement adheres to our policy

The datasets used in the analyses for this study are freely available to download from the following locations: MODIS land surface temperature data: https://

www.earthdata.nasa.gov/; European Centre for Medium-Range Weather Forecasts Reanalysis v5 (ERA5) Analysis-Ready, Cloud Optimized (ARCO) dataset: https://github.com/google-research/arco-era5; Global Forest Change (GFC) dataset: https://storage.googleapis.com/earthenginepartners-hansen/GFC-2023-v1.11/download.html; Global Land Analysis and Discovery (GLAD) Global Land Cover and Land Use Change dataset: https://glad.umd.edu/dataset/GLCLUC2020; Global Multi-resolution Terrain Elevation Data (GMTED2010): https://earthexplorer.usgs.gov/; LandScan population data: https://landscan.ornl.gov/; Global Human Settlement Layer dataset: https://human-settlement.emergency.copernicus.eu/ghs_smod2023.php; Global Burden of Disease (GBD) Study cause-specific mortality rates: https://gbd2019.healthdata.org/gbd-results/ or https://vizhub.healthdata.org/gbd-results/. Processed data files produced and used in the analyses of this study will be available through Code Ocean upon acceptance (provisional DOI: 10.24433/CO.9925363.v1; Python code used to produce the central results of this study will be available as a Compute Capsule through Code Ocean upon acceptance (provisional DOI: 10.24433/CO.9925363.v1; https://codeocean.com/capsule/9925363/tree).).

Research involving human participants, their data, or biological material

Policy information about studies with human participants or human data. See also policy information about sex, gender (identity/presentation), and sexual orientation and race, ethnicity and racism.

Reporting on sex and gender	N/A - No human research participants were involved in this study
Reporting on race, ethnicity, or other socially relevant groupings	N/A
Population characteristics	N/A
Recruitment	N/A
Ethics oversight	N/A

Note that full information on the approval of the study protocol must also be provided in the manuscript.

Field-specific reporting

Please select the one below that is the best fit for your research. If you are not sure, read the appropriate sections before making your selection.

☐ Life sciences ☐ Behavioural & social sciences ☒ Ecological, evolutionary & environmental sciences

For a reference copy of the document with all sections, see nature.com/documents/nr-reporting-summary-flat.pdf

Ecological, evolutionary & environmental sciences study design

All studies must disclose on these points even when the disclosure is negative.

Study description	The study used preexisting satellite-derived datasets so no data collection was performed for this study.
Research sample	N/A
Sampling strategy	N/A
Data collection	N/A
Timing and spatial scale	N/A
Data exclusions	N/A
Reproducibility	N/A
Randomization	N/A
Blinding	N/A

Did the study involve field work? ☐ Yes ☒ No

Reporting for specific materials, systems and methods

We require information from authors about some types of materials, experimental systems and methods used in many studies. Here, indicate whether each material, system or method listed is relevant to your study. If you are not sure if a list item applies to your research, read the appropriate section before selecting a response.

Materials & experimental systems

n/a	Involved in the study
☒	☐ Antibodies
☒	☐ Eukaryotic cell lines
☒	☐ Palaeontology and archaeology
☒	☐ Animals and other organisms
☒	☐ Clinical data
☒	☐ Dual use research of concern
☒	☐ Plants

Methods

n/a	Involved in the study
☒	☐ ChIP-seq
☒	☐ Flow cytometry
☒	☐ MRI-based neuroimaging

Plants

Seed stocks	N/A
Novel plant genotypes	N/A
Authentication	N/A
